# Treatment of early borderline lesions in low immunological risk kidney transplant patients: a Spanish multicenter, randomized, controlled parallel-group study protocol: the TRAINING study

**DOI:** 10.1186/s12882-022-02989-z

**Published:** 2022-11-07

**Authors:** Domingo Hernández, Teresa Vázquez-Sánchez, Eugenia Sola, Veronica Lopez, Pedro Ruiz-Esteban, Abelardo Caballero, Eduardo Salido, Myriam Leon, Aurelio Rodriguez, Nuria Serra, Consuelo Rodriguez, Carme Facundo, Manel Perello, Irene Silva, Domingo Marrero-Miranda, Ignacio Cidraque, Francesc Moreso, Luis Guirado, Daniel Serón, Armando Torres

**Affiliations:** 1 Nephrology Department, Hospital Regional Universitario de Málaga and University of Málaga, IBIMA, REDinREN (RD16/0009/0006 and RICORS RD21/0005/0012), E-29010 Málaga, Spain; 2 Nephrology Department, Carlos Haya Regional University Hospital, Avda. Carlos Haya s/n., E-29010 Malaga, Spain; 3 Immunology Department, Hospital Regional Universitario de Málaga and University of Málaga, IBIMA, REDinREN (RD16/0009/0006 and RICORS RD21/0005/0012), E-29010 Malaga, Spain; 4 Pathology Department, Hospital Universitario de Canarias, Instituto de Tecnologías Biomédicas-Universidad La Laguna, E-38320 Tenerife, Spain; 5 Pathology Department, Hospital Regional Universitario de Malaga, IBIMA, REDinREN (RD16/0009/0006 and RICORS RD21/0005/0012), E-29010 Málaga, Spain; 6 Nephrology Department, Hospital Universitario de Canarias, Instituto de Tecnologías Biomédicas-Universidad La Laguna, REDinREN (RD16/0009/0031), E-38320 Tenerife, Spain; 7 Nephrology Department, Fundación Puigvert, E-08025 Barcelona, Spain; 8 Nephrology Department, Hospital Universitari Valld’Hebron and Universitat Autonoma, REDinREN (RD16/0009/0030 and RICORS RD21/0005/0016), E-08035 Barcelona, Spain

**Keywords:** kidney transplant, borderline lesions, α-klotho, low immunological risk, subclinical inflammation

## Abstract

**Background:**

Subclinical inflammation, including borderline lesions (BL), is very common (30–40%) after kidney transplantation (KT), even in low immunological risk patients, and can lead to interstitial fibrosis/tubular atrophy (IFTA) and worsening of renal function with graft loss. Few controlled studies have analyzed the therapeutic benefit of treating these BL on renal function and graft histology. Furthermore, these studies have only used bolus steroids, which may be insufficient to slow the progression of these lesions. Klotho, a transmembrane protein produced mainly in the kidney with antifibrotic properties, plays a crucial role in the senescence-inflammation binomial of kidney tissue. Systemic and local inflammation decrease renal tissue expression and soluble levels of α-klotho. It is therefore important to determine whether treatment of BL prevents a decrease in α-klotho levels, progression of IFTA, and loss of kidney function.

**Methods:**

The TRAINING study will randomize 80 patients with low immunological risk who will receive their first KT. The aim of the study is to determine whether the treatment of early BL (3rd month post-KT) with polyclonal rabbit antithymocyte globulin (Grafalon®) (6 mg/kg/day) prevents or decreases the progression of IFTA and the worsening of graft function compared to conventional therapy after two years post-KT, as well as to analyze whether treatment of BL with Grafalon® can modify the expression and levels of klotho, as well as the pro-inflammatory cytokines that regulate its expression.

**Discussion:**

This phase IV investigator-driven, randomized, placebo-controlled clinical trial will examine the efficacy and safety of Grafalon® treatment in low-immunological-risk KT patients with early BL.

**Trial registration:**

clinicaltrials.gov: NCT04936282. Registered June 23, 2021, https://clinicaltrials.gov/ct2/show/NCT04936282?term=NCT04936282&draw=2&rank=1. Protocol Version 2 of 21 January 2022. Sponsor: Canary Isles Institute for Health Research Foundation, Canary Isles (FIISC). mgomez@fciisc.org.

## Background

Short-term outcomes after kidney transplantation (KT) have improved significantly, but 30% of grafts lose their function within 10 years of transplantation [1]. The predominant histological lesion is a combination of inflammation and interstitial fibrosis/tubular atrophy (IFTA) [2–5]. Subclinical inflammation, including borderline lesions (BL), is very common after KT (30–40%), even in patients with low immunological risk and stable graft function. These lesions can lead to IFTA, worsening of graft function and loss of the KT, especially when interstitial inflammation is associated with tubulitis [6–10]. However, there is lack evidence of treating BL lesions and slowering allograft function. A recent randomized study by our group (ClinicalTrials.gov, number NCT02284464), performed in recipients with a low immunological risk with and without steroids, showed that those who had BL at the third month post-KT presented more IFTA and worse renal function at 24 months post-KT compared to patients without inflammation, which was more prevalent in those who did not receive steroids [11].

There is evidence that not treating these lesions can lead to chronic histological changes and impaired graft function [10, 12], but most previous studies were not controlled. Additionally, they did not focus on treatment for subclinical inflammation that did not reach the score for acute rejection ≥IA (i2,t2) of the Banff’19 classification [13]. Moreover, they mostly only used bolus steroids, which could be insufficient to slow the progression of these lesions [14, 15]. Therefore, the clinical impact of adequate anti-rejection treatment remains controversial [7, 16]. Controlled studies are needed to assess the therapeutic response in patients with BL to improve graft survival.

Polyclonal rabbit antithymocyte globulins (Grafalon® and Thymoglobulin®) are very effective for the prevention and treatment of immunological dysfunction post-KT [17, 18]. Grafalon® (Neovii Pharmaceuticals AG) has a higher clearance compared to Thymoglobulin® (Sanofi Genzyme) and is associated with a faster reconstitution of the lymphocytic subpopulations (CD3, CD4 and CD8) and generation of regulatory T cells than Thymoglobulin® [19, 20]. Grafalon® could represent an effective therapeutic tool to suppress or minimize the early tubulo-interstitial lymphocytic infiltrate with few infectious complications. This should prevent long-term worsening of kidney graft function.

Finally, Klotho is a transmembrane protein produced mainly in the kidney that has antifibrotic and antiapoptotic properties, playing a crucial role in the aging-inflammation binomial of kidney tissue and progression of chronic kidney disease (CKD) [21, 22]. In fact, α-klotho deficiency in animal models and in human studies is associated with early senescence, kidney fibrosis and accelerated arteriosclerosis [23, 24]. Systemic and local inflammation decrease renal tissue expression and soluble levels of α-klotho [25]. Likewise, proinflammatory cytokines (TNF-α or TWEAK) induce less expression of α-klotho, contributing to tissue damage [26]. Therefore, it is plausible that a sustained subclinical inflammation of the grafts could contribute to the reduction in α-klotho and premature graft senescence.

It remains to be clarified whether anti-rejection treatment of BL, detected early in the third month post-KT, could prevent the decrease in α-klotho levels, the progression of IFTA and the loss of kidney function. Accordingly, it is reasonable to undertake a randomized controlled clinical trial to assess the evolution of early BL and levels of α-klotho after anti-rejection therapy with Grafalon®. Determining a favorable therapeutic response would provide valuable clinical information to improve the long-term survival of KT in patients with low immunological risk.

## Methods/Desing

### Objectives

#### Primary objectives

Determine whether treatment of BL, detected at the third month post-KT, with Grafalon® prevents or reduces the progression of IFTA and the deterioration of graft function compared to conventional clinical follow-up, after two years post-KT.

#### Secondary objectives

1. Analyze the incidence of immunologic dysfunction (acute clinical and subclinical rejections) and the histological evolution.
2. Determine whether anti-rejection therapy of BL can modify klotho expression and levels and the pro-inflammatory cytokines that regulate its expression.
3. Assess the association between α-klotho, urinary chemokine levels (CXCL9 and CXCL10), and acute clinical and subclinical rejection, as well as with the IFTA lesions.
4. Detect the generation of donor-specific anti-HLA antibodies (DSA).
5. Determine the impact of anti-rejection treatment on proteinuria during follow-up.
6. Record graft and patient survival at the first and second year of follow-up.
7. Assess the adherence to immunosuppressive therapy and its impact on IFTA lesions and kidney function.

### Trial Design

This is a Spanish multicenter, investigator-driven, randomized, open-label, two-arm, clinical trial with a 24-month follow-up in patients with a first deceased donor or living donor KT who have BL at the time of the third-month protocol biopsy. The immunosuppressants in the study arms are those routinely used in clinical practice. This is a phase IV clinical trial under the usual conditions (clinicaltrials.gov: NCT04936282). Patient recruitment time will be 12 months. The minimum follow-up period for patients will be-come 2 years, when a second protocol biopsy will be performed.

### Eligibility Criteria and Exclusions

#### Inclusion criteria

- Patients of either sex, older than 18 years, with low immunological risk (PRA < 20% and absence of DSA), who receive their first deceased donor or living donor KT.
- Presence of BL, excluding isolated inflammation (t0, i > 0) and isolated tubulitis (t > 0, i0)
- Patients receiving tacrolimus in combination with mycophenolic acid (MPA) and steroids.
- Absence of clinical or subclinical and histological inflammation-related allograft dysfunction before randomization
- Absence of de novo DSA anti-HLA antibodies at the time of randomization.
- Provision of written informed consent (obtained by the attending physician).
- Acceptance of efficient contraception in women.

#### Exclusion criteria

- Recipients of a multi-organ transplant.
- Re-transplants
- Patients with biopsy-proven acute rejection or no histological inflammation.
- Cold ischemia time > 30 hours.
- Serum creatinine >2 mg/dl or proteinuria >1 g/day at randomization.
- Presence of significant thrombopenia (<100,000/mm3) or leukopenia (<3000 mm/3) at randomization.
- Previous episode of clinical or subclinical rejection (≥IA) before randomization.
- Presence of BL before randomization
- CMV infection or disease in the first three months after transplantation
- BK-polyomavirus nephropathy at randomization.
- Recurrent or de novo glomerulonephritis.
- Treatment with immunosuppressive drugs other than those in this clinical trial.
- Patients who are positive for the human immunodeficiency virus or with severe systemic infection, who, in the opinion of the investigator, require continued therapy.
- Previous (within the last 5 years) or present malignancy, except excised basal or squamous cell carcinoma
- Pregnant or lactating women.

#### Subject withdrawal criteria

If the study is interrupted for any reason, the patient will be considered to have discontinued treatment prematurely and will only be included in the “intention to treat” (ITT) analysis and will be eliminated from the “per protocol” (PP) analysis.

All subjects who are excluded from the study for any reason will continue with the same follow-up as those who remain in the protocol to perform a more robust analysis by intention to treat.

### Study Procedures and Work Plan

This study will be carried out in four Spanish transplant centers (Hospital Regional Universitario de Málaga; Hospital Universitario de Canarias, Tenerife; Hospital Vall d’Hebron, and Hospital Fundacio Puigvert, Barcelona) within the Kidney Research Network (RICORS).

Any modification to the protocol will be approved by the relevant Ethics and Research Committee and the Spanish Drug Agency and notified as necessary.

### Randomization

Subjects will be randomized to one of the arms in a 1:1 ratio, carried out locally in each center through the electronic data collection notebook (e-DCN) that will include a computer program for the generation of random numbers. If a patient is randomized but does not receive the study treatment, they will not be included in either the ITT or the PP analysis. Their number and treatment group will not be reused for new patients.

### Treatment Arms

#### Study Arms: control (Group I)

- Tacrolimus: Advagraf® 0.15 mg/kg/day or Envarsus® (0.1 mg/kg/day) to maintain levels of 8–12 ng/ml at the first month. Thereafter Advagraf® 0.1 mg/kg/day or Envarsus® 0.07 mg/kg/day to maintain levels of 6–8 ng/ml.
- Mycophenolate mofetil (MMF) 2 g/day or mycophenolic acid (MPA) 1440 mg/day the first 30 days post-KT. Afterwards MMF 1 g/day or MPA 720 mg/day.
- Steroids: 0.5 g Methylprednisolone pre-KT and 125 mg on day 1; prednisone 30 mg the first 15 days post-KT and then 5 mg/week until reaching 5 mg/day at the 2nd month post-KT and the same dose will be continued.

This treatment will continue until randomization.

#### Study Arms: Intervention (Group II)

Apart from the standard treatment taken in Group I, Group II will receive treatment with Grafalon® as follows.

- Grafalon® 6 mg/kg/day in a single day. Its administration will be done as follows:

- Pre-medication before the first and second doses: Solumoderin® 125 mg i.v. and antihistamine H1 antagonist (dexchlorpheniramine [Polaramine® 1 vial (5 mg) i.v.], diphenhydramine, or other from the same therapeutic group).
- Grafalon®: Dilute the calculated dose in 500 ml of physiological serum and administer within 8 hours through a central line or A-V fistula.
- Administer half the dose if leukocytes <3000/mm3 and platelets <80,000/mm3. Stop when leukocytes <1500/mm3 or platelets <40,000/mm3.
- During treatment with Grafalon®:
  - MMF/MPA: 500 mg/12 h or 360 mg/12 h, monitoring leukocyte and platelet counts.
  - Anti-CMV (except in the case of negative IgG recipient and donor) and Pneumocystis prophylaxis must be given.

DSA will be monitored (every 3 months in the first year and every 6 months in the second year). Additionally, immunosuppression will be maintained throughout the study (post-randomization) following the therapeutic schedule:

- Steroids 5 mg/day
- Maintain levels of tacrolimus (8–10 ng/ml) to achieve a proper immunosuppression during study.
- MMF doses of at least 1 g/day or MPA 720 mg/day
- Do not administer anti-mTOR drugs

In both therapeutic arms, induction treatment with anti-CD25 monoclonal antibodies (Basiliximab 20 mg i.v., days 0 and 4) or polyclonal antibodies (Thymoglobulin 1 mg/kg/day for 4–7 days) will be allowed in patients at risk of delayed graft function.

Patients without inflammation (i0,t0) at the third month protocol biopsy, or with just isolated inflammation (i > 0,t0) or isolated tubulitis (t > 0,i0), will continue conventional immunosuppression (steroids+Tac + MMF) and be monitored until study end, performing the protocol biopsy at month 24, in order to determine the natural history of the histological changes and the evolution of renal function, comparing them to patients with BL and randomized to the two therapeutic arms.

Immunosuppressants will be prescribed and administered according to routine clinical practice, so their packaging and labeling is approved for marketing.

#### Other treatments

Acute rejection will be treated with 3 boluses of 500 mg/day of methylprednisolone i.v. Corticosteroid resistant rejections will be treated with a standard 7–10 day cycle of thymoglobulin. Acute humoral rejections will be treated according to the protocol of each center, whether it is plasmapheresis, immunoglobulins, and rituximab, alone or in combination.

Drugs to prevent the most frequent infections such as Pneumocystis jirovecii and CMV will be used according to the protocol of each center. Dyslipidemia will be treated with statins, or with ezetimibe when adverse effects appear. Fibrates should be avoided, with the exception of gemfibrozil, as they can be associated with impaired kidney function. Hypertension will be treated according to the protocol of each center to obtain a target BP <130/80 mmHg. The treatment of post-transplant diabetes will be carried out according to the usual therapeutic indications of this entity.

#### Safety committee and Safety monitoring

This is composed of those responsible for each center. Periodically and via teleconference, adverse effects will be analyzed in both study groups. Interim safety analysis is preplanned by the sponsor during study, to the independent data monitoring and safety committee.

In addition, the sponsor may end the study at any time and for any reason.

### Definitions of end points

Primary end points: Presence of IFTA and graft function at 24 months in both therapeutic arms.

Main efficacy end points: renal function and histological lesions.

- The glomerular filtration rate (GFR) determined by CKD-EPI formula.
- Appearance of acute or chronic pathological lesions on allograft biopsy. Biopsies will be performed at the third month post-transplantation and at month 24 post-randomization. They will be evaluated according to the Banff’19 classification [13]. For a correct interpretation of the biopsies, at least 10 glomeruli and 2 artery will be required.

The different histological diagnostic categories will be:

1. No inflammation (i0,t0)
2. BL, defined as:

- Foci of tubulitis (t1,t2,t3) with mild inflammation (i1), or mild tubulitis (t1) with moderate-to-severe inflammation (i2 or i3), but below the threshold for Banff IA rejection (i2,t2). Therefore, the possible combinations of BL scores are: i1t1, i2t1, i3t1, i1t2 and i1t3.
- Absence of transmural arteritis (v0)

Due to the excellent prognosis of lesions with isolated inflammation (i > 0, t0) [10] and isolated tubulitis (i0, t > 0) [27], patients with these lesions will not be included in the randomized arms of the study.

A graft biopsy will be performed at any time during the study if graft dysfunction is detected, evidenced by proteinuria ≥0.5 g/day or an increase in serum creatinine ≥25% over baseline value. Biopsies for clinical indication and those done pre-randomization will be assessed in each participating center. Local pathologist interpretation will be performed to analyze the presence of clinical or subclinical rejection, BL or the progression of chronic lesions that suggest an eventual graft dysfunction. Biopsies corresponding to the pre-randomization and final visits will also be sent to the central pathologist at the Hospital Universitario de Canarias, who will give a second interpretation based on the same Banff/19 criteria.

The score sum of the classification of chronic lesions will be obtained through 5 histological characteristics:

- cg: chronic glomerulopathy
- ci: chronic interstitial
- ct: tubular atrophy (chronic)
- cv: arteriolar hyalinosis (chronic)
- mm: mesangial matrix

Chronic injuries will be defined as IFTA scores ≥2 (ci + ct) according to the Banff’19 classification. The score sum of all chronicity compartments (cg + ci + ct + cv) will also be taken into account.

Secondary efficacy variables:

a) Graft and patient survival.

b) Biopsy-proven acute rejection rate, and proteinuria at 3,6,12 and 24 months.

c) Blood pressure (BP) and number of hypotensive drugs, lipid levels and need for lipid-lowering drugs, weight gain and body mass index.

d) Post-transplant diabetes rate or glucose intolerance according to ADA criteria [28].

e) Adherence to immunosuppressants: evaluated using the BAASIS scale at each visit of the study by interview [29].

-Serum levels of α-klotho will be measured by ELISA using a specific kit for soluble human α-klotho (Immuno-Biological Lab.). This assay has an inter- and intra-assay coefficient of variability of 5% and 5.0–7.3%, respectively, and a lower limit of detection of 3.0 relative units/ml. Klotho will also be measured in urine [25]. Circulating FGF-23 levels will be measured using an ELISA kit for human (C-terminal) FGF-23 (Immunotopics). Plasma levels of soluble TNF-alpha and TWEAK/Fn14 will also be determined by ELISA with a specific kit [30, 31].

-Urinary chemokines CXCL9 and CXCL10 will be determined with an immunoassay kit (Milliprex®). The intra-assay coefficient of variation is <10%, and the inter-assay <15%.

#### Methods and schedule for evaluating efficacy parameters

**Fig. 1:**
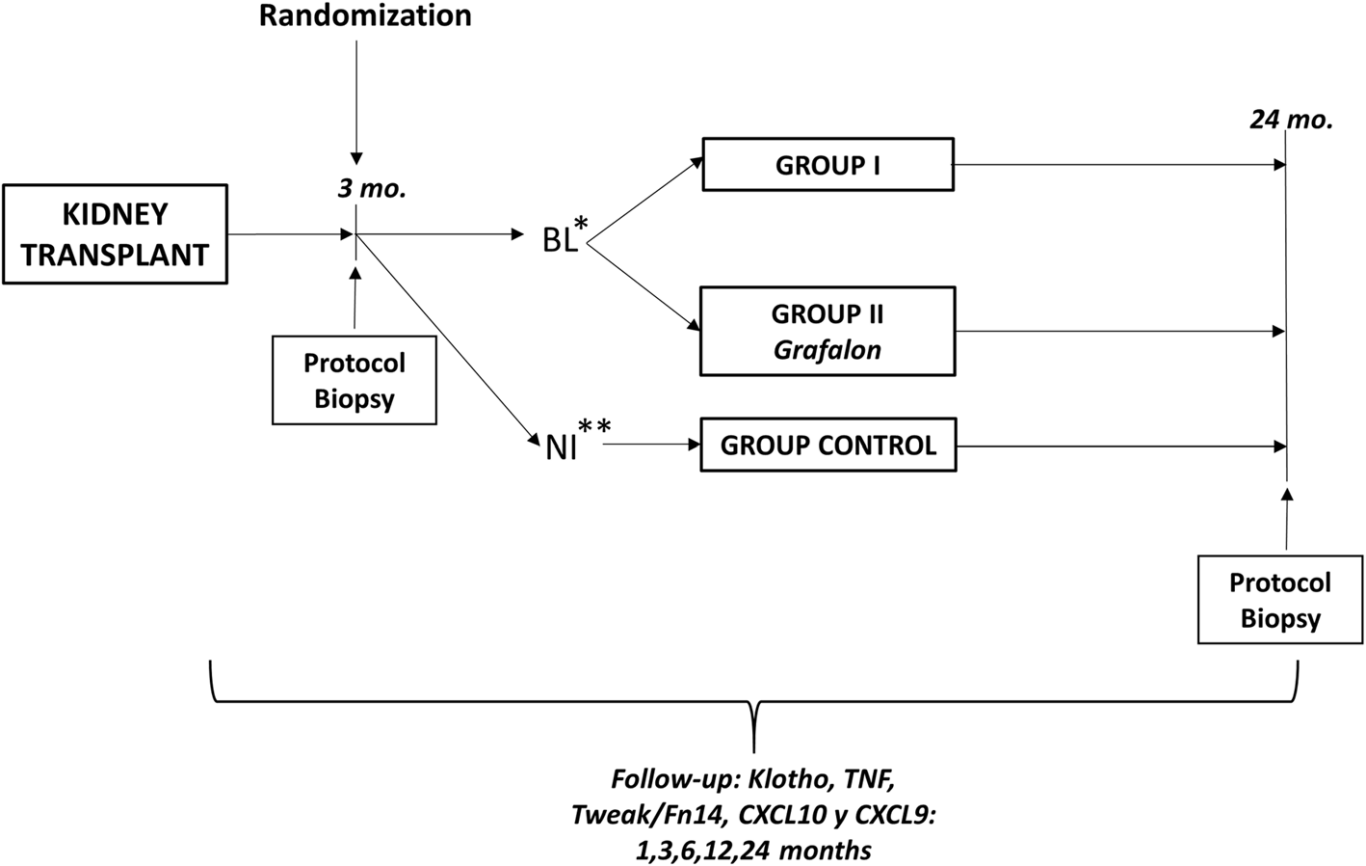
Scheme and determinations of the study

The study schedule is shown in Tables 1 and 2. Figure 1 displays the scheme and determinations of the study.

**Table 1:** Study visits and evaluation schedule

Visit	Baseline KT	Month 1	Month 2 Pre-randomization Screening	Month 3 Randomization	Month 4	Month 6	Month 9	Month 12	Month 18	Month 24
	1	2	3	4	5	6	7	8	9	10
**Informed consent**	**X**									
**Clinical and demographic data**	**X**	**X**	**X**	**X**	**X**	**X**	**X**	**X**	**X**	**X**
**Pregnancy test**			**X**	**X**						
**Randomization criteria**			**X**	**X**						
**Clinical incidents (hypertension, diabetes, dyslipidemia)**	**X**	**X**	**X**	**X**	**X**	**X**	**X**	**X**	**X**	**X**
**Kidney graft biopsy**				**X**						**X**
**General lab tests**^a^	**X**	**X**	**X**	**X**	**X**	**X**	**X**	**X**	**X**	**X**
**Anti-HLA (DSA) antibodies**				**X**				**X**		**X**
**Renal function** **(CKD-EPI)**		**X**	**X**	**X**	**X**	**X**	**X**	**X**	**X**	**X**
**Proteinuria**		**X**	**X**	**X**	**X**	**X**	**X**	**X**	**X**	**X**
**Urinary chemokines (CXCL9 and CXCL10)**		**X**		**X**		**X**		**X**		**X**
**Elisa Kits: Klotho, FGF-23 TNF and TWEAK/Fn14**		**X**		**X**		**X**		**X**		**X**
**Assessment of therapeutic compliance (BAASIC scale)**				**X**				**X**		**X**

^a^blood glucose, Calcium, creatinine, Phosphorus, Hematocrit, Hemoglobin, HDL-Cholesterol, LDL-Cholesterol, parathyroid hormone, tacrolimus levels, Total Cholesterol, triglycerides

**Table 2:** Visiting schedule

**Activity**	**Visits**										
	**0**	**1**	**2**	**3**	**4**	**5**	**6**	**7**	**8**	**9**	**10**
**Donor demographic data**		**X**									
**Graft characteristics**		**X**									
**Recipient clinical and demographic data**		**X**									
**• Clinical and laboratory data:** **• Anthropometric parameters** **• Delayed graft function** **• Anticalcineurin levels** **• Fasting blood glucose and hypoglycemic treatment** **• Serum creatinine** **• Estimated filtration (CKD-EPI)** **• Ultrasensitive C-reactive protein (PCR)** **• Acute rejection** **• Data** ○ **Histological Type (Banff Classification ‘19)** ○ **Treatment received** ○ **Corticosensitive/Corticoresistant** ○ **Serum creatinine at the start of treatment** **• BP, total cholesterol, HDL-cholesterol and LDL-cholesterol values** **• Proteinuria: protein / creatinine ratio or 24-hour proteinuria** **• Infections** **• Medical and surgical complications** **• Concomitant drugs**			**X**	**X**	**X**	**X**	**X**	**X**	**X**	**X**	**X**
**Informed consent**		**X**									
**Protocol biopsy**					**X**						**X**
**Anti-HLA antibodies**		**X**		**X**		**X**			**X**	**X**	**X**
**Assessment of therapeutic compliance**				**X**					**X**		**X**

#### Safety assessment

All adverse events (AEs) will be monitored during follow-up.

The Safety Committee is composed of those responsible at each participating center. A project safety report will be drawn up every six months. The Safety Committee will analyze whether is it safe to continue with the study based on the cumulative number of cases.

The requirements of Spanish Royal Decree 1090/2015 (article 51) on the notification of adverse reactions will be followed. In particular, any serious adverse event should be reported to the electronic Case Report Form (eCRF) within 24 hours and reported to all participating centers.

Required data for the analysis of AEs will be registered by the investigator in an eCRF using a real-time data recording system “on-line”.

#### Data monitoring, management, access and dissemination

Data monitoring will be undertaken by the Spanish Clinical Research Network (SCReN) in collaboration with Health Institute Carlos III. Data will be recorded by each centre in the general database, and access will be controlled by Spanish law (Royal Decree 1090/2015). The results will be communicated through the usual channels.

### Data analysis and statistics

#### Sample size

Data analyzed in previous studies where renal function is compared in patients with BL versus those without inflammation have been used as reference points [32, 33]. To detect a difference in GFR of 10 ml/min at 24 months post-KT in both groups with an estimated standard deviation in one of the groups of 15 ml/min, a power of 80% (Beta = 0.20), and a statistical risk of 5% (alpha = 0.05), a total of 70 patients with BL (35 in each therapeutic arm) is required. Assuming a 15% loss to follow-up, 80 patients (40 in each group) are needed for the evaluation of efficacy (Epidat 3.1).

#### Statistical analysis

Descriptive statistics will be presented with the statistical analyses. An intention-to-treat and end-treatment analysis will be performed. Categorical variables will be analyzed according to the chi-square test or Fisher’s exact test. Quantitative data will be analyzed using the Student’s t-test or the Mann-Whitney U test. In the case of three quantitative samples, ANOVA or Kruskal-Wallis will be used as appropriate. Changes in the evolution over time of renal function in the two study groups will be analyzed using the general linear model for repeated measures, adjusting for confounding variables. Graft and patient survival curves will be calculated by the Kaplan-Meier method. The survival rates will be compared between groups with the log-rank test method. A linear regression analysis will be done to determine the factors associated with changes in kidney function and the progression of chronic lesions. Data will be analyzed using SPSS 20.0.

## Discussion

This study will provide information on the progression of IFTA and the relationship between klotho levels and subclinical inflammation (specifically BL) in patients with a low immunological risk, evidenced at the 3-month protocol biopsy. We will also assess other proinflammatory molecules and chemokines related with the inflammation-senescence binomial, furthering our understanding of the pathogenic mechanisms that lead to accelerated graft senescence when subclinical inflammation is present. This study can contribute to establishing a new therapeutic approach in these patients, in order to improve long-term post-KT outcomes. It will allow us to compare the natural history of the histological changes of patients without inflammation with those with early BL included in the therapeutic arms.

Group I patients could have a higher risk of acute rejection when not receiving Grafalon®. However, there is no consensus on the ideal treatment of BL detected in protocol biopsies, nor are there sufficient controlled clinical trials to conclusively demonstrate that not treating BL lesions can trigger acute immune dysfunction [7, 16]. In any case, the risk of acute rejection and the generation of DSA can be minimized if we consider that only subjects with a low immunological risk will be included and will receive as induction anti-CD25 (Basiliximab) or polyclonal antibodies (Thymoglobulin) in case of a higher risk of delayed graft function. In addition, anti-HLA antibodies will be closely monitored and a series of therapeutic measures will be carried out aimed at minimizing immunological risk throughout the study. Moreover, we will closely monitor immunosuppression-related side effects such as diabetes and life-threatening complications (cardiovascular disease or infections) [28].

This study will be carried out at four Spanish transplant centers with intense transplant activity, which guarantees reaching the required sample size. In addition, Spain has the highest donor and transplant rate in the word. Thus, a sufficient number of KT with early BL is expected in our study. Although the information will be taken from records of each hospital, which could lead to information bias, the exhaustive follow-up and the collection of information in a centralized electronic platform should minimize this concern.

In conclusion, this Spanish multicenter, investigator-driven, randomized, placebo-controlled phase IV clinical trial will assess the efficacy and safety of treatment of early BL with polyclonal rabbit antithymocyte globulin in low immunological risk KT patients.

## Data Availability

Not applicable.

## Abbreviations

AEs: Adverse events
BL: Borderline lesions
BP: Blood pressure
CKD: Chronic kidney disease
DSA: Donor-specific anti-HLA antibodies
eCRF: Electronic Case Report Form
GFR: Glomerular filtration rate
IFTA: Interstitial fibrosis/tubular atrophy
ITT: Intention to treat
KT: Kidney Transplantation
MMF: Mcophenolate mofetil
MPA: Mycophenolic acid
PP: Per protocol
